# Digital technology and national entrepreneurship: An ecosystem perspective

**DOI:** 10.1007/s10961-022-09934-0

**Published:** 2022-05-17

**Authors:** Jianhong Zhang, Désirée van Gorp, Henk Kievit

**Affiliations:** Nyenrode Business Universiteit, Straatweg 25, 3621 BG Breukelen, The Netherlands

**Keywords:** Digital technology, National entrepreneurial ecosystems, Supportive culture, Institutional quality, Resource endowments

## Abstract

Although the importance of digital technology has been recognized in the entrepreneurship literature, we know relatively little about how and to what extent it influences a nation’s entrepreneurial activities. Drawing on the concept of entrepreneurial ecosystem, this study developed a conceptual model to explain the impact of digital technology on national entrepreneurship and the interactions between digital technology and other ecosystem elements. The hypotheses are tested by using unbalanced panel data of 101 countries from 2001 to 2018. The empirical results show that the level of digital technology is positively associated with the output of national entrepreneurial ecosystems, and this positive relationship is strengthened in nations with a supportive culture, high-quality institutions, supportive policies, accessible resources, and well-developed service industries. The findings highlight the importance of digital technology, provide fresh insights into the interdependence between elements and causal mechanisms in national entrepreneurial ecosystems.

## Introduction

It has been widely acknowledged that entrepreneurship is essential for a region’s or country’s development (Acs and Armington 2004; Huggins and Williams 2011; Schumpeter 1934). However, how to promote entrepreneurship remains a continuing question. In recent years, entrepreneurial ecosystems have become increasingly popular in explaining high-growth entrepreneurship and the interaction between entrepreneurs and their environments (Audretsch, Cunningham, et al. 2019; Autio, Nambisan, et al. 2018; Colombo et al. 2019; Feldman et al. 2019; Spigel 2017; Vedula and Kim 2019; Volkmann et al. 2019). Spigel (2017) indicated that a successful entrepreneurial ecosystem leads to high rates of entrepreneurship. Entrepreneurship or entrepreneurial activities have been considered the output of the ecosystem (Stam 2015; Stam and Spigel 2016).

New digital technologies have, over decades now, dramatically transformed the world economy and the way of doing business, which is accelerated by the COVID-19 pandemic (UNCTAD 2021). Digital technologies and their applications have begun to reshape the nature and structure of organizations and to change the economic landscape (Burtch et al. 2018; Kallinikos 2007; Katz and Koutroumpis 2013; Nambisan et al. 2017; Nambisan, Wright, et al. 2019; Nambisan, Zahra, et al. 2019; Yoo et al. 2012). Specifically, digital technologies are changing the way firms produce, market and distribute goods and services and contribute to economic growth and living standards. In turn, these changes are opening up a broader set of opportunities for entrepreneurs to exploit (Nambisan 2017; Zahra and Nambisan 2011). In this sense, digital technology is considered an external enabler of entrepreneurial activities (von Briel et al. 2018), among other enablers such as culture, institutions, and demand (Davidsson 2015), playing an important role in entrepreneurial ecosystems (Autio, Nambisan, et al. 2018; Elia et al. 2020). Despite its importance, research has paid little attention to the impact of digital technology on entrepreneurship (Elia et al. 2020) and there is a lack of empirical evidence to support the idea that digital technology helps to promote entrepreneurship in a country or region. Entrepreneurial ecosystem studies have recognized that digital technology enhances the connection between different ecosystem actors (Bouncken and Kraus 2021), and changes the nature of interactions among actors in the business ecosystem (Elia et al. 2020; Zahra and Nambisan 2011). However, the role of digital technology in the ecosystem is under-researched (Song 2019).

Literature on entrepreneurial ecosystems highlights the interdependence among the elements. It indicates that the knowledge of how these elements are interrelated explains the overall functioning of the entrepreneurial ecosystem (Wurth et al. 2021). Despite studies have provided several theoretical frameworks to reveal interdependencies between the elements (Motoyama and Knowlton 2017; Spigel 2017; Stam and van de Ven 2021; Wurth et al. 2021), “it is not always clear in what way proposed elements are connected” (Alvedalen and Boschma 2017, p. 897). Besides, most studies on this topic have been largely theoretical or case-based because of the novelty of the concept of entrepreneurial ecosystems (Wurth et al. 2021). There is a lack of empirical findings to validate the interdependencies and causal mechanisms.

In addition, the boundaries of an (entrepreneurial) ecosystem are not well defined in the literature (Audretsch, Cunningham, et al. 2019; Colombo et al. 2019; Parente et al. 2019). Some studies argue ecosystems are a regional-level (or city-level) phenomenon because the entrepreneurial process depends on learning and knowledge and resource exchanges in regional contexts (e.g., Audretsch, Belitski, et al. 2019; Spigel and Harrison 2018; Stam 2015). However, digitalization (i.e., the process of adopting digital technologies) reduces the dependency of new ventures on a specific location for entrepreneurial opportunities. It facilitates the opportunity pursuit beyond a region by alleviating the spatial constraints regarding knowledge and market access (Autio, Nambisan, et al. 2018; Voelker et al. 2017). While digitalization blurs the regional boundaries separating different entrepreneurial ecosystems, the national boundary becomes more relevant especially when considering the policy implications. Entrepreneurial activities are “regulated by contextual factors, such as culture, formal institutions and resource availability” (Acs et al. 2014, p. 481). These factors are considered elements of entrepreneurial ecosystems (Stam and Spigel 2016), and in most cases, they are national attributes (Autio et al. 2014). Although digitalization may enable the ecosystem boundary to go beyond a country border (i.e., cross-country ecosystem), national boundaries of culture, resources and regulations remain essential. From the perspective of national policymakers, it is important and necessary to identify system-level bottlenecks and alleviating them (Acs, Audretsch, et al. 2016). The research on national entrepreneurial ecosystems fits this need. Therefore, investigating entrepreneurial ecosystems as a national phenomenon can enrich the literature and draw theoretical and policy implications. However, national entrepreneurial ecosystems are not sufficiently researched.

In response, this study addresses these gaps by answering two research questions: to what extent does digital technology influence national entrepreneurship, and how is this influence conditional on other elements in the entrepreneurial ecosystem? To answer these questions, we create a conceptual model based on the entrepreneurial ecosystem frameworks developed by Spigel (2017) and Stam and van de Ven (2021). Despite these frameworks being mainly developed at a regional level, they can be aggregated on and applicable to a country level because the elements are not region- or city-specific. In the conceptual model, we consider digital technology an element of the ecosystem. We argue that digital technology can influence the output of the national entrepreneurial ecosystem by interaction with other elements. We apply a cross-country research design that takes a nation as an entrepreneurial ecosystem (Acs et al. 2014; Audretsch, Cunningham, et al. 2019). We use longitudinal data of 101 countries over 17 years (2001–2018) to examine our hypotheses.

Answering these research questions, we contribute to the literature by introducing digital technology into the national entrepreneurial ecosystem as a new element while investigating the interdependency between the elements. We do not empirically verify the ecosystem development and the interdependence of all elements in the ecosystem. A disclosure is made to what extent digital technology influences the system output by interacting with other elements in the entrepreneurial ecosystem. The findings advance the understanding of the interdependencies within the ecosystem and can inform national policy development on system-level bottlenecks. The theoretical and policy implications are discussed in more detail in the last section.

The remainder of this paper is structured as follows. Followed by this introduction, the next section provides the conceptual framework and hypotheses. Thereafter, the variables, sample, and estimation technics are described. Then, the results are presented. In the last subsequent section, the theoretical contribution and practical implications for policymakers are drawn.

## Theoretical Background and Hypotheses

This section develops a conceptual model to explain how digital technology and other elements jointly explain national entrepreneurship development from an entrepreneurial ecosystem perspective. We first review the literature and set up the conceptual model, then we develop our baseline hypothesis regarding the effect of digital technology on the system-level output of the national entrepreneurial ecosystem, and the hypotheses regarding the interdependency/interaction between digital technology and the elements that belong to three commonly addressed entrepreneurial ecosystem components, culture, formal institutions, and resource endowments.

### Entrepreneurial ecosystems

Understanding interdependencies between elements is crucial for understanding the entrepreneurial ecosystems (Spigel 2017; Wurth et al. 2021). Spigel (2017) proposed a pyramid model, demonstrating that the lower components support higher components and higher components reinforce lower components. The interactions between the components create dynamics of the ecosystem. Stam and van de Ven (2021) proposed an integrative model of entrepreneurial ecosystems consisting of ten elements and entrepreneurial outputs, emphasizing the presence of these elements and the interdependence between them are crucial for the success of the ecosystem. Based on an extensive literature review, Wurth et al. (2021) developed a comprehensive model to demonstrate the causal relationships among the ecosystem elements (intra-layer causation), how the elements lead to outputs and outcomes (the upward causation), and how outcomes and outputs feedback into the system conditions (downward causation). These advancements in the ecosystem studies imply that without knowing the interdependencies we can hardly understand the role of each element in the entrepreneurial ecosystem.

To uncover these interdependencies, we need to know what comprises an entrepreneurial ecosystem. Studies proposed several frameworks to describe the elements of entrepreneurial ecosystems. Spigel (2017) suggested that ecosystems are composed of eleven attributes, namely supportive culture, histories of entrepreneurship worker talent, investment capital, networks, mentors and role models, policy and governance, universities, support services, physical infrastructure, and open markets. Similarly, Stam and van de Ven (2021) indicated that the ecosystem includes the institutional arrangements (i.e., the formal institutions, culture and network elements) and resource endowments (i.e., the physical infrastructure, finance, leadership, talent, knowledge, intermediate services and demand elements). Corrente et al. (2019) investigated the importance of the entrepreneurial ecosystem factors and found that the most important factors could be identified in cultural and social norms, government programs, and internal market dynamics. These studies all addressed elements that belong to the three fundamental components: culture, formal institutions, and resource endowments.

Besides the above-mentioned elements, recent studies pay more attention to the role of technologies. Audretsch et al. (2019) articulated three key elements, namely the technological, economic, and societal dimensions of ecosystems, positing technology as a parallel element as economic and societal ones. Autio et al. (2018) proposed a structural model of entrepreneurial ecosystems, highlighting the importance of digitalization. The model illustrates that the combination of digital and spatial affordances facilitates “business model innovation for entrepreneurial opportunity discovery and pursuit that, in turn, characterizes entrepreneurial ecosystems” (Autio, Nambisan, et al. 2018, p. 83). Digital affordances are the action potential or possibilities offered by digital technology (Nambisan, Wright, et al. 2019); while, spatial affordances are the spatial mechanisms that facilitate and regulate economic activity (Autio, Nambisan, et al. 2018). Nambisan et al. (2019) extended this model by introducing social affordances (Sileno et al. 2014) and institutional affordances (van Dijk et al. 2011) into the analytical framework. They called for research efforts to investigate the interaction effects among these affordances to understand how similar initiatives in different contexts lead to different outcomes. They advocated to “explore how the interactions between digital affordances and institutional affordances (at the level of national and regional governments) help stimulate and coordinate innovation and entrepreneurial ecosystems” (Nambisan, Wright, et al. 2019, pp. 4–5).

In response, we take digital technology as one of the elements and investigate how digital technology and other elements jointly reproduce the entrepreneurial ecosystem. Our literature review above indicates that there are three commonly recognized entrepreneurial ecosystem components, namely culture, formal institutions, and resource endowments. We, therefore, explore how elements in the three sets of components strengthen the effect of digital technology. Culture, as the “software of the mind” (Hofstede 1991), forms the base of the ecosystem, because institutional and economical components are all connected to norms and values of how things ought to be done. Formal institutions are the rules of a game that are humanly devised to shape human interactions (North 1990). While formal institutions are embedded in cultural settings, they regulate individual and organizational actions such as resource distributions and exchanges and entrepreneurial activities. Resource endowments refer to the resources that are necessary to facilitate entrepreneurial activities. They are closely linked to a nation’s economic conditions.

**Fig. 1:**
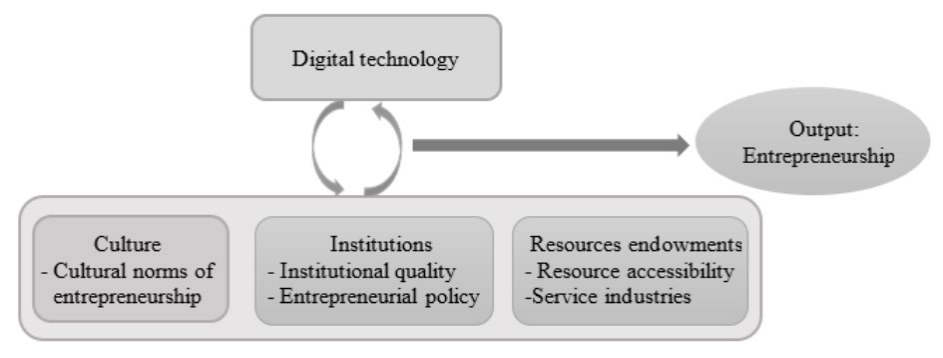
Conceptual model

Based on the entrepreneurial ecosystem perspective, we argue that digital technology as a newly developed element influences the output of the national entrepreneurial ecosystem by interacting with other elements. In our empirical analysis, we focus on the interaction between digital technology and other elements, which means we tend to uncover the relationships in which the effect of digital technology on ecosystem output depends on the state of other elements. By doing so, we reveal causal mechanisms in entrepreneurial ecosystems. Figure 1 presents our conceptual model, indicating the elements and their interactions investigated in the national entrepreneurial ecosystem.

### Digital technology and entrepreneurial ecosystems

Nambisan (2017, p. 1031) indicated that “digital technologies manifest in the realm of entrepreneurship in the form of three distinct but related elements: digital artifacts, digital platforms, and digital infrastructure.” Digital technology enables firms to adopt new ways of doing business that significantly transform innovation and entrepreneurship (Nambisan 2017; Nambisan et al. 2017; Nambisan, Wright, et al. 2019; Yoo et al. 2010). Accordingly, various studies have argued that digital technology should be taken as a key explanatory factor when theorizing on the nature and process of entrepreneurship (Kenney and Zysman 2016; Nambisan 2017; Nambisan, Wright, et al. 2019). In this study, we do not intend to take stock of research on the relationship between digital technology and entrepreneurship, instead, we focus on the phenomena related to entrepreneurial ecosystems. Recent studies proposed a new framework, the digital entrepreneurial ecosystem (DEE), to explain the role that digital technology plays in entrepreneurship (Du et al. 2018; Elia et al. 2020; Song 2019; Sussan and Acs 2017). In a DEE, digital technology is considered a condition (Autio, et al. 2018; Torres and Godinho 2021) because the DEE focuses on a specific type of entrepreneurship, digital entrepreneurship. Therefore the DEE is a subset of the entrepreneurial ecosystem (Sussan and Acs 2017), which may not reveal the general role of digital technology in an entrepreneurial ecosystem. This study aims to investigate the role of digital technology by considering it as an element in the entrepreneurial ecosystem. Our logic is as follows. Digital technology connects people, things, and locations, which means that digital technology plays an intermediary role that enhances the connections between the elements in the ecosystem (Bouncken and Kraus 2021). This intermediary role is similar to those of intermediaries, one element in the entrepreneurial ecosystem (Stam and van de Ven 2021; Wurth et al. 2021). In addition, digital technologies in form of digital platforms and digital infrastructure can be considered as a type of infrastructure, which is another element of the entrepreneurial ecosystem (Stam and van de Ven 2021; Wurth et al. 2021). Furthermore, technologies in general together with other entrepreneurial ecosystem elements (i.e., culture, institutions and demand) are considered enablers of entrepreneurial activities (Davidsson 2015). Given its importance and specialty, we take digital technology as a basic element in the national entrepreneurial ecosystem.

In their conceptual model of entrepreneurial ecosystems, Autio et al. (2018) explained the effect of digital technology on entrepreneurship by introducing the concept of digital affordance. They argued that digitalization supports three key digital affordances: decoupling (reducing the importance of asset specificity); disintermediation (reducing the power of middlemen in value chains); and generativity (enabling the coordination of geographically dispersed audiences). These affordances empower entrepreneurs to find new ways to create, deliver and capture value, thereby discovering and pursuing entrepreneurial opportunities. Therefore, digital technology facilitates entrepreneurial ecosystem output.

Applying this logic to the national entrepreneurial ecosystem, we propose our baseline hypothesis as:

H1. The level of digital technology in a country is positively associated with entrepreneurship in that country.

### Interaction between digital technology and other societal and economic elements of the national entrepreneurial ecosystem

#### The role of culture

Culture is an important reflection of a country’s informal institutions, representing shared values and noncodified standards (Holmes et al. 2013; North 1990). Culture determines how people think, believe and behave, regulates their approach to relationships with others (De Clercq et al. 2013), and determines their occupation choice (e.g., choosing to be an entrepreneur) (Light and Dana 2013). In the context of entrepreneurship, we focus on the underlying beliefs and attitudes about entrepreneurial behavior (Spigel 2017). This cultural attribute is a crucial factor that affects the perception of entrepreneurship desirability and, consequently, influences entrepreneurial intention and behavior (Ajzen and Fishbein 1980; Krueger Jr and Brazeal 1994; Veciana et al. 2005). Here, we explain how this cultural attribute may suppress or enhance the effect of digital technology on entrepreneurship.

Although digital technology may induce more entrepreneurial opportunities, the application of digital technology is deeply intertwined with regional/national cultures, since culture shapes acceptable entrepreneurial practices and norms (Aoyama 2009). Various studies have articulated that digitalization releases some traditional conditions of doing business, creating more potential for entrepreneurial opportunities (Autio, Nambisan, et al. 2018; Nambisan 2017). However, the opportunities are exploited by some entrepreneurs, but not by others. In an environment where entrepreneurial behavior is encouraged, individuals and organizations can easily gain social assistance to exploit new resources, such as open resources, digital platforms and digital infrastructure to support their new business activities. Hence, the effect of digital technology on entrepreneurship is strengthened. However, in an environment where entrepreneurial behavior is not encouraged, potential opportunities derived from digital technology may not be well-explored. This means that the effect of digital technology on entrepreneurship is conditional on entrepreneurship culture. At the same time, digital technology may also change people’s perception of businesses, hence influence entrepreneurship culture. For example, digital technology helps to spread success stories, good experiences and positive effects of entrepreneurial activities. This, in turn, supports the development of a culture supportive of entrepreneurship. It is a reinforcing effect that further enhances the interaction relationship. From an entrepreneurial ecosystem perspective, this iterative interaction leads to high output, which is entrepreneurial activities.

Applying the logic to the national entrepreneurial ecosystem, we propose the following hypothesis:

H2. Digital technology and culture interact such that the positive effect of digital technology on entrepreneurship is strengthened in countries with a culture supportive of entrepreneurship.

#### The role of formal institutions

Institutions contain the constraints and the incentives that are humanly devised to shape human interaction. Institutions include formal and informal constraints. The formal include, e.g., political rules, economic rules and contracts; and the informal constraints vary from unwritten codes of conduct, norms to values and conventions (North, 1990).

This section focuses on formal institutions. It is defined as “the rules of game” (North, 1990) that constrain, enable and guide entrepreneurial activity (Aidis et al. 2008; Welter and Smallbone 2011). Well-developed institutions, clarifying the order of a transaction process, lower transaction costs by alleviating uncertainty (Child and Rodrigues 2011) and facilitate the development of entrepreneurship (Welter and Smallbone 2011). The direct relationship between institutions and entrepreneurship has been discussed previously in the literature (e.g., Aidis et al. 2008; Klapper et al. 2006; Thornton et al. 2011; Welter 2011; Welter and Smallbone 2011). We extend this research by investigating how institutions and digital technology interact and influence entrepreneurship.

While digital technology provides new entrepreneurial opportunities (Autio, Nambisan, et al. 2018) uncertainties may hinder entrepreneurs from starting a new business (Matthews and Scott 1995; Mckelvie et al. 2011). Formal institutions, such as rules and policies, are established to reduce uncertainty about the activities of organizations by standardizing practices and demanding conformance (Holmes et al. 2013). In a well-developed institutional environment uncertainty and risk are relatively low where there are transparent regulatory systems, sufficient legal protection, and service support that provide a good environment for entrepreneurs with new ventures (Batjargal et al. 2013; Welter and Smallbone 2011). This pro-business environment encourages entrepreneurs to explore new business opportunities emerging in the process of adopting digital technologies. However, in a weak institutional environment, there is a lack of sound regulations to facilitate market transactions. This generates uncertainty and ambiguity for new businesses and an environment discouraging entrepreneurs from exploring new opportunities provided by digital technology. Therefore, the effect of digital technology on entrepreneurship is conditional on institutional quality. At the same time, digital technology over time may also influence formal institutions, as institutional theory predicts that technology development causes institutional change (North 1990). For example, data science, digital marketing, digital platforms and cybersecurity pose new challenges to existing formal institutions because they change the relationships and transactions where these institutions were set (e.g., Boon et al. 2019). The challenges push authorities to improve formal institutional quality to meet the needs of business development in the digital era (Wolfe 2021). From an entrepreneurial ecosystem perspective, the iterative interaction between digital technology and formal institutions leads to high output. Therefore, we expect that digital technology and institutional quality jointly influence the national entrepreneurial ecosystem output. Accordingly, we propose:

H3. Digital technology and formal institutions interact such that the positive effect of digital technology on entrepreneurship is strengthened in countries with high-quality formal institutions.

Besides the general quality of institutions, the role of the specific institutions related to entrepreneurship also needs to be clarified. This study focuses on the entrepreneurial policy that “represent laws and directives that create publicly-funded support programs designed to encourage entrepreneurship through tax benefits, investment of public funds, or reductions in bureaucratic regulation” (Spigel 2017, p. 54). Although the effectiveness of entrepreneurial policy regarding the social and economic consequences (e.g., employment, growth rate and innovation) is debatable (Arshed et al. 2014; Lerner 2012; Mason and Brown 2013; Shane 2009), the policy remains an element of the entrepreneurial ecosystem (Spigel 2017). Effective policy solutions are needed to encourage entrepreneurship and sustainable and resilient entrepreneurship-led economic growth (Grilli 2004; Spigel and Harrison 2018).

Entrepreneurship is associated with uncertainty because it involves new business models, new products, and new ideas (Drucker 1986; Magnani and Zucchella 2018). While new technologies (e.g., digital technologies) help to create entrepreneurial opportunities, their novelty also creates risk and uncertainty (Mckelvie et al. 2011). The risk and uncertainty discourage entrepreneurs from identifying and utilizing the opportunities derived from digital technology. In this case, external support such as government supports may help entrepreneurs overcome the risk and uncertainty associated with the new business. The policy designed to facilitate entrepreneurial activities, such as tax reliefs, loans or subsidies, or reductions in bureaucratic regulation, meets this need. Therefore, in countries with effective entrepreneurial policies, entrepreneurs are more likely to identify and utilize the potential opportunities presented by digital technology because it helps to provide the resources needed and to make the digital-driven activities more resilient and sustainable. On the contrary, in countries without effective entrepreneurial policies, entrepreneurs may avoid exploiting new technology-based opportunities because of the challenges associated with new entrepreneurial activities. Therefore, the effect of digital technology on entrepreneurship depends on the availability of entrepreneurial policies. At the same time, entrepreneurial policies, as a part of formal institutions, may also be influenced by digital technology. Noticing that digital technology has vastly changed the business landscape, policymakers in many countries see the necessity to adjust their entrepreneurial policy to respond to the changes presented by digital technology (Thomas et al. 2019). This feedback effect further enhances the interaction relationship that leads to high entrepreneurial ecosystem output. Based on these arguments, we propose:

H4. Digital technology and entrepreneurial policy interact such that the positive effect of digital technology on entrepreneurship is strengthened in countries with supportive entrepreneurial policies.

#### The role of resource endowments

Existing frameworks have addressed the resource endowments in the entrepreneurial ecosystem. In Spigel’s model, the economic resources such as workers’ talents, investment capital, support services and open market access, are stated (Spigel 2017, p. 57). In the framework of Stam and van de Ven (2021), resources such as physical infrastructure, finance, leadership, talent, knowledge, intermediate services and demand elements are presented. In our empirical analysis, we focus on two elements related closely to digital technology: physical resources accessibility and the development of the service industry.

Physical resources include communication, utilities, transportation, land or space (Hindle 2010). The accessibility of physical resources influences the likelihood that entrepreneurs explore the potential technology-based opportunities derived from digital technology. In a country where physical resources are easy to access by entrepreneurs, they are more willing to start a new business by using the potential opportunities derived from digital technology. On the contrary, if entrepreneurs cannot access the physical resources in a country, entrepreneurial activities are hindered. This means that the effect of digital technology on entrepreneurship depends on the accessibility of physical resources in a country. At the same time, digital technology improves the accessibility of physical resources because the process of adopting digital technology in business is interrelated with physical resources (Gann et al. 2011). This feedback effect further enhances the interaction relationship that leads to high national entrepreneurial ecosystem output. Based on this argument, we propose:

H5. Digital technology and physical resources interact such that the positive effect of digital technology on entrepreneurship is strengthened in countries where physical resources are easy to access.

Services are an element in the entrepreneurial ecosystem (Spigel 2017). In a broad sense, the service industry plays an important role in a country’s economic advancement (Buera and Kaboski 2012). Advanced service sectors are often associated with more economic opportunities for business (Thai and Turkina 2014). The service industry includes both public and private sectors. A well-developed public service sector provides good national public services, such as education, healthcare and social security, and the private counterpart provides supporting business services such as technical services, legal services, employment services, logistics services and facility management. All of these services facilitate entrepreneurial activities (Ben Youssef et al. 2021; Newman and Clarke 2009; Spigel 2017). In a country where the service industry is well developed, entrepreneurs are more likely to start new businesses by using the potential opportunities derived from digital technology. However, if the service industry is not well developed, entrepreneurs cannot access the services needed, then the new opportunities provided by digital technology may not be materialized. This means that the effect of digital technology on entrepreneurship depends on the development of the service industry in a country. At the same time, digital technology facilitates the development of a nation’s service industry (Maiti and Kayal 2017). For example, digital technology facilitates service innovation (Barrett et al. 2015). This feedback effect further enhances the interaction relationship that leads to high national entrepreneurial ecosystem output. Based on thses arguments, we propose:

H6. Digital technology and service industry interact such that the positive effect of digital technology on entrepreneurship is strengthened in countries where the service industry is well developed.

## Method

### Variables

Dependent variable.

*Entrepreneurship* measures the system-level output of the national entrepreneurial ecosystem. The Global Entrepreneurship Monitor (GEM) ‘Adult Population Survey’ (APS) is used to construct this variable. APS is a unique instrument used to measure the level of entrepreneurial activity worldwide (Nicotra et al. 2018). Following the approach of Acs et al. (2014), we use a measurement that covers entrepreneurial activities, and perception, abilities, and aspiration (Acs et al. 2018). It is measured by four items from APS. One item measures entrepreneurial activity (total early-stage entrepreneurial activities), and the other three items measure entrepreneurial perceptions, abilities, and aspiration, namely: perceived opportunities rate, perceived capabilities rate, and entrepreneurial intentions rate. The four items are chosen because, on the one hand, they reflect a nation’s entrepreneurial building blocks (pillars) (Acs et al. 2018). On the other hand, GEM provides the most completed data for these four items cross countries and years. The factor scores of the four items are used to measure this variable. The factor loadings and Cronbach’s Alpha are presented in Table 1.

**Table 1:** The reliability assessment of Entrepreneurship and Entrepreneurial policy

Variables	Items	Loadings	Cronbach’s Alpha
Entrepreneurship	Perceived opportunities rate	0.786	0.848
	Perceived capabilities rate	0.873	
	Entrepreneurial intentions rate	0.876	
	Early-stage entrepreneurial activity rate	0.904	
Entrepreneurial policy	Financing for entrepreneurs	0.744	0.849
	Governmental support and policies	0.878	
	Taxes and bureaucracy	0.818	
	Governmental programs	0.885	

Key explanatory variable.

*Digital* measures the application level of digital technology at the national level, indicating the extent to which digital technology is used. In the realm of entrepreneurship, digital technologies occur in the form of three distinct, but related, elements: digital artifacts, digital platforms, and digital infrastructure (Nambisan 2017). We use the number of mobile users per 100 inhabitants to measure the application of digital artifacts, and the number of internet users per 100 inhabitants to measure digital platforms and digital infrastructure. These two indicators are often used in the literature to measure the application of digital technology (Bagchi 2005; Chinn and Fairlie 2007; Park et al. 2015; Rath 2016). We use the number of internet users in our main estimation and the number of mobile users, as an alternative measure in the robustness tests.

Moderators.

Moderators in this study refer to the variables that capture the elements that belong to the three components (culture, formal institutions, and resource endowments) in the ecosystem. We use them to investigate the interaction effects proposed in our hypotheses.

*Cultural norm* denotes the entrepreneurship culture of a nation in a specific year. It measures the extent to which social and cultural norms encourage or allow actions leading to entrepreneurial activities. We use one item (i.e., cultural and social norms) from the GEM database to measure it.

*Institutional quality* denotes the institutional quality of a nation in a specific year. The data are derived from the World Bank’s Worldwide Governance Indicators (WGI). The WGI index has been widely used in the literature to measure institutional quality (Dwumfour and Ntow-Gyamfi 2018; Law et al. 2014; Lu et al. 2014). The WGI index is composed of six indicators: voice and accountability, political stability, government effectiveness, regulatory quality, rule of law, and control of corruption. Given the high correlation between these six indicators, this study follows previous research measuring formal institutional quality by averaging these six indicators into a single broader index (He and Zhang 2018).

*Entrepreneurial policy* denotes the entrepreneurial policy of a nation in a specific year. It is measured by four items from the GEM database. The four items (i.e., financing for entrepreneurs, governmental support and policies, taxes and bureaucracy, and governmental programs) reflect the extent to which a nation supports entrepreneurial activities. The factor scores are used to measure this variable. The factor loadings and Cronbach’s Alpha are presented in Table 1.

*Resource accessibility* denotes the accessibility of the physical resources of a nation in a specific year. It is measured by one item from the GEM, the ease of access to physical resources.

*Service industry* indicates the development of the service industry of a nation in a specific year. It is measured by the share of the service industry in a nation’s GDP. The data are retrieved from the World Development Indicators (WDI).

Control variable.

We include the following variables in our estimation models that have been identified in the literature as being relevant to entrepreneurship.

*Capital* denotes the richness of financial capital in a nation in a specific year. Financial capital is a basic condition for starting a new business (Cetindamar et al. 2012; Schwienbacher 2007). It is measured by the ratio of gross capital formation to GDP in host countries. The high ratio shows a greater abundance of financial capital in a country. The data are derived from the WDI.

*Education* denotes entrepreneurial education in a nation in a specific year. The literature documents a linkage between entrepreneurial education and entrepreneurship outcomes (for a detailed review see Martin, Mcnally and Kay, 2013). This variable indicates the extent to which training in creating or managing SMEs is incorporated within the education and training system at school. The high value indicates a high possibility of receiving entrepreneurial education. The data are derived from the GEM.

*Market* denotes internal market changes. Studies have documented a relationship between market change and entrepreneurship (Corrente et al. 2019; Farinha et al. 2020; Martínez-Fierro et al. 2016). This variable is measured by an item from the GEM, Internal Market Dynamics. The high value indicates a high level of change in internal markets.

*Trade* denotes the importance of international trade in a nation. The reason to include this variable is that international trade extends market boundaries and provides entrepreneurs with opportunities to access global markets and foreign inputs (Bjørnskov and Foss 2008; Grossman 1984). It is measured by the ratio of trade to GDP of a nation in a specific year. The data are derived from the WDI.

### Sample and estimation techniques

The sample was derived from the GEM Adult Population Survey (APS). It is the largest multi-country research project on entrepreneurship providing individual and country-level harmonized data on entrepreneurial orientation, attitudes, activities, and business characteristics^1^. It has been widely used to study entrepreneurship (Brieger et al. 2020; De Clercq et al. 2013; Hörisch et al. 2017, 2019; Jha and Bhuyan 2019; Klyver et al. 2008; Stuetzer et al. 2014; Young et al. 2018). We use a longitudinal unbalanced panel design and a panel data set across 110 countries over the period 2001–2018 (N = 771).

We use a fixed effect panel data model in our estimations to address potential omitted variable bias related to unobserved national characteristics. A Hausman test (chi-square = 552.03, p-value < 0.001) also suggests that the fixed effects model is more appropriate than the random-effects model.

To test the interaction between the national entrepreneurial ecosystem elements, we use the approach recommended by Brambor, Clark, and Golder (2006). The strength of this approach is that it can avoid overstating and understating an interaction effect by evaluating not only the coefficient of the interaction term but also the marginal effect of one variable over the range of values of another variable (Kingsley et al. 2017).

We checked for potential multicollinearity problems before conducting model estimations by calculating the correlations between the independent variables. Table 2 shows that all the correlation coefficients are lower than the commonly used threshold of 0.7. In addition, the variance inflation factors (VIF) values for each model used are calculated. The results show that the values are far less than the cut-off level of 10. We can conclude that multicollinearity is not a problem in the used models.

**Table 2:** Correlation matrices

		1	2	3	4	5	6	7	8	9	10	11
1	Entrepreneurship	1										
2	Digital	-0.435*	1									
3	Capital	0.059	-0.114*	1								
4	Education	-0.057	0.246*	0.118*	1							
5	Market	-0.091*	-0.087*	0.236*	0.045	1						
6	Trade	-0.254*	0.201*	0.047	0.243*	0.072	1					
7	Cultural norms	0.195*	0.091*	0.159*	0.485*	0.088*	0.010	1				
8	Institutional quality	-0.510*	0.684*	-0.150*	0.282*	-0.276*	0.251*	0.035	1			
9	Entrepreneurial policy	-0.249*	0.444*	0.209*	0.490*	-0.059	0.308*	0.442*	0.541*	1		
10	Resource accessibility	-0.328*	0.534*	0.075	0.213*	-0.112*	0.341*	0.231*	0.538*	0.538*	1	
11	Service industry	-0.435*	0.663*	-0.358*	0.147*	-0.326*	0.099*	0.090*	0.683*	0.282*	0.385*	1

*significant at 0.05

### Results

The results of the fixed effect estimation are presented in Table 3. Model 1 shows the results with control variables and moderators. Model 2 adds the key variable *Digital* measured by the number of internet users per 100 inhabitants. Models 3–7 further include the five interaction terms separately: Digital*Cultural, Digital*Institutional quality, Digital*Entrepreneurial policy, Digital*Resource accessibility, Digital*Service industry.

**Table 3:** Estimation result

	Model 1	Model 2	Model 3	Model 4	Model 5	Model 6	Model 7
Digital		0.007***	0.007***	0.009***	0.008***	0.007***	0.006***
		(0.001)	(0.001)	(0.002)	(0.002)	(0.001)	(0.001)
Capital	0.015***	0.016***	0.016***	0.016***	0.014***	0.016***	0.017***
	(0.005)	(0.005)	(0.005)	(0.005)	(0.005)	(0.005)	(0.005)
Education	-0.078	-0.061	-0.077	-0.060	-0.093	-0.062	-0.047
	(0.072)	(0.071)	(0.071)	(0.070)	(0.071)	(0.071)	(0.071)
Market	-0.017	-0.021	-0.021	-0.019	-0.016	-0.014	-0.022
	(0.027)	(0.026)	(0.026)	(0.025)	(0.026)	(0.026)	(0.026)
Trade	0.005***	0.005***	0.006***	0.004***	0.005***	0.005***	0.005***
	(0.001)	(0.001)	(0.001)	(0.001)	(0.001)	(0.001)	(0.001)
Cultural norms	0.151**	0.107	0.089	0.084	0.094	0.080	0.087
	(0.068)	(0.067)	(0.067)	(0.066)	(0.066)	(0.067)	(0.067)
Institutional quality	0.001	0.004	0.003	0.008	0.004	0.005	0.004
	(0.005)	(0.005)	(0.005)	(0.005)	(0.005)	(0.005)	(0.005)
Entrepreneurial policy	-0.025	-0.031	-0.025	-0.034	-0.026	-0.032	-0.031
	(0.034)	(0.034)	(0.034)	(0.033)	(0.033)	(0.033)	(0.033)
Resource accessibility	-0.051	-0.073	-0.068	-0.047	-0.067	-0.067	-0.046
	(0.057)	(0.055)	(0.055)	(0.055)	(0.055)	(0.055)	(0.056)
Service industry	0.024***	-0.004	-0.004	-0.014	-0.012	-0.006	0.000
	(0.006)	(0.009)	(0.009)	(0.009)	(0.009)	(0.009)	(0.009)
Digital*Cultural norms			0.003**				
			(0.001)				
Digital*Institutional quality				0.000***			
				(0.000)			
Digital*Entrepreneurial policy					0.002***		
					(0.001)		
Digital*Resource accessibility						0.005***	
						(0.002)	
Digital*Service industry							0.0002***
							(0.000)
Constant	-2.245***	-1.042	-0.889	-0.760	-0.522	-0.932	-1.384**
	(0.583)	(0.654)	(0.657)	(0.650)	(0.670)	(0.651)	(0.661)
Observations	771	755	755	755	755	755	755
R-squared	0.059	0.101	0.107	0.124	0.115	0.114	0.113
Number of code	101	100	100	100	100	100	100
Log likelihood test	-201.5	-165.6	-163.2	-156	-159.8	-160.3	-160.8

Standard errors in parentheses

***p < 0.01, **p < 0.05, *p < 0.1

Models 1 and 2 examine the direct impact of the key explanatory variable on *Entrepreneurship*. Comparing the two models, we see the R-squared increases significantly when *Digital* is included. The coefficient (B = 0.007; p < 0.01) is positive and significant. This result supports H1.

**Fig. 2:**
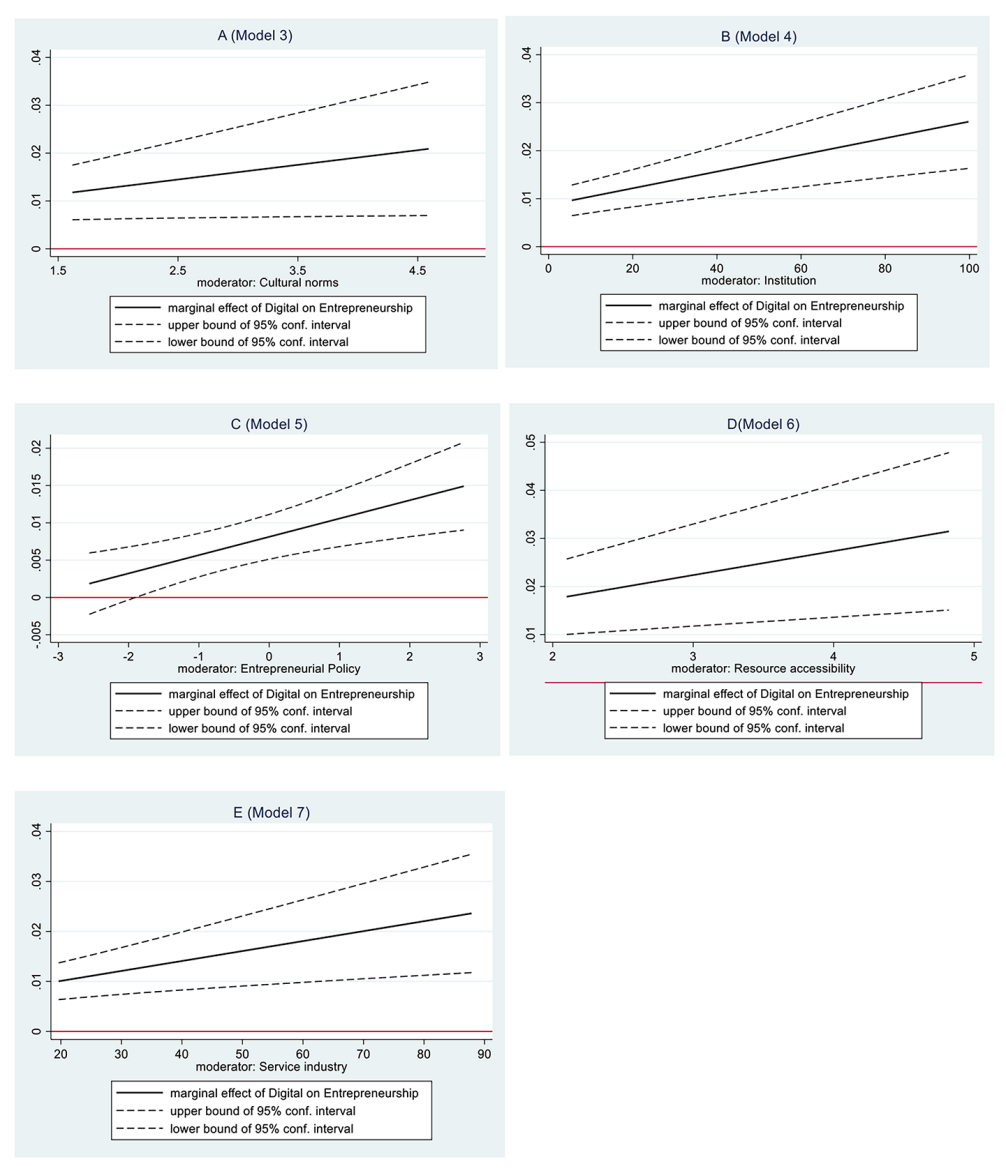
Moderating Effect of the five moderators on the relationship between digital technology and entrepreneurship

Models 3–7 examine the interaction effects. The coefficients of the five interaction terms are positive and significant, Digital*Cultural (B = 0.024, p < 0.05), Digital*Institutional quality (B = 0.001, p < 0.01), Digital*Entrepreneurial policy (B = 0.002, p < 0.01), Digital*Resource accessibility (B = 0.005, p < 0.01), Digital*Service industry (B = 0.0002, p < 0.01). This result demonstrates that there are positive interactions between *Digital* and the five moderators respectively. To avoid the overestimating problem (Kingsley et al. 2017), the marginal effects of *Digital* on *Entrepreneurship* under the conditions of the five moderators are calculated by using the variance and covariance obtained from Models 3–7. The results presented in Fig. 2 show that the marginal effect of *Digital* on *Entrepreneurship* is significant across the entire range of *Cultural norms*(A), *Institutional quality* (B), *Resource accessibility* (D), and *Service industry* (E), the major range (98.4%) of *Entrepreneurial policy* (C). This result confirms the interaction effects of digital technology with five elements and supports H2-6.

### Robustness test

We performed several analyses to look at the robustness of the results.

First, we estimated the models by using an alternative measure of the independent variable - the number of mobile users per 100 inhabitants. This measure is complementary to the number of internet users used in the main analysis. The number of mobile users gauges the application of digital artifacts. The number of internet users assesses the utilization of digital platforms and digital infrastructure. We estimated the same specifications as in our main analysis.

Second, we used the start-up rate to measure the dependent variable. It was measured by the percentage of 18–64 age group in the population who are either nascent entrepreneurs or owner-managers of new businesses. This measure was included as one of the items of our composed measurement in our main analysis. We used it alone here as an alternative measure of the dependent variable because it is often used in the literature to measure entrepreneurship (e.g., Audretsch and Keilbach 2007; Stuetzer et al. 2014).

Third, we examined the robustness of our results by eliminating the influential observations. In our country sample, the United States could be an influential one because of its leading role in digital technology (European Commission 2020). Therefore, we excluded observations of the United States and estimated the same specifications as in our main analysis.

Fourth, we added three extra variables to control the impact of the external changes on entrepreneurship. We added two dummies (2008 and 2009) to control the effect of the possible influence of the global crisis in 2008. Unemployment also may influence entrepreneurship (for a detailed review see Baptista and Thurik, 2007). We added the unemployment rate in the estimations.

The outcomes of these four extra analyses are presented in Appendixes 1–4 and produce results consistent with the main estimations presented in Table 3.

## Discussion

This study investigates the relationship between digital technology and entrepreneurship by using a unique multisource panel data set across 110 countries. The results indicate that national digital technology is positively associated with national entrepreneurship. The findings confirm that digital technology can be considered an element of the national entrepreneurial ecosystem in the digital era. The results also indicate that the contribution of digital technology to the output of the national entrepreneurial ecosystem is conditional on cultural, institutional, and economic elements. Specifically, we find that the positive relationship between digital technology and entrepreneurship is intensified under the conditions that a nation has a supportive culture, high-quality institutions, supportive entrepreneurial policies, accessible physical resources and well-developed service industries. This finding confirms the theoretical prediction that interactions between elements of a national entrepreneurial ecosystem improve the entrepreneurial performance of a nation (Alvedalen and Boschma 2017; Mack and Mayer 2016), disclosing some causal mechanisms that drive the development of entrepreneurial ecosystems (Wurth et al. 2021).

Interestingly, the results of this study indicate that the following five elements, *Cultural norms, Institutional quality, Entrepreneurial policy, Resource accessibility*, and *Service industry*, are statistically insignificant. This implies that favorable cultural, institutional and economic conditions do not lead directly to a high level of entrepreneurship. This finding is in line with the previous studies documenting that some favorable contextual factors do not have a positive effect on entrepreneurship. For example, De Clercq et al. (2013) found that an institutional factor (financial system) has a negative impact and a culture factor (trust) does not have a significant effect on entrepreneurship. Jha and Bhuyan (2019) found that institutional aspects, such as government stability, law and order, and judiciary independence, all have an insignificant effect on entrepreneurship. The impact of the entrepreneurial policy is also debatable. Acs et al. (2016) reviewed the established evidence and found that most of the entrepreneurial policies fail to promote valuable entrepreneurship. These findings lead us to argue that, although elements of the national entrepreneurial ecosystem provide benefits and resources to entrepreneurs, one element alone may not foster entrepreneurship directly. Instead, the relationships between these elements reproduce the entrepreneurial ecosystem (Stam and Spigel 2016). To understand the effect of one element, we need to analyze the conditions or complementary factors that make the elements effective.

### Theoretical implication

This study contributes to existing entrepreneurship research in several ways.

First, this study extends the literature on entrepreneurial ecosystems by clarifying the important role of digital technology. We argue that, besides the elements discussed in the literature (i.e. culture, formal institutions, and resource endowments) (Spigel 2017; Stam and van de Ven 2021), digital technology can be viewed as an important element of the entrepreneurial ecosystem in the digital era. Although a few studies have discussed the role of digital technology, they have focused on a specific ecosystem, the DEE (Du et al. 2018; Elia et al. 2020; Song 2019; Sussan and Acs 2017; Torres and Godinho 2021). The DEE is a subset of the two larger systems: the digital ecosystem and the entrepreneurial ecosystem (Sussan and Acs 2017). Therefore, this system, by definition, is a part of an entrepreneurial ecosystem composed of heterogeneous digital entities (Li et al. 2012; Sussan and Acs 2017). However, as digital technology penetrates all sectors, it should be defined as a fundamental pillar in the broader system i.e., (national) entrepreneurial ecosystem, not only in a specific subset.

Second, this study advances our understanding of the entrepreneurial ecosystem by uncovering the interaction between the ecosystem elements. Although it has been well documented that the interdependence between the elements facilitates value creation and strengthens an ecosystem and leads to high entrepreneurial output (e.g., Acs et al. 2017; Audretsch, Cunningham, et al. 2019; Spigel 2017), studies are largely at a conceptual level, and more solid empirical evidence is required (Neumeyer et al. 2019). The findings of this study address this gap by showing that digital technology, as an element of the entrepreneurial ecosystem, plays an important role in the ecosystem. More importantly, digital technology contributes to the entrepreneurial ecosystem output by interacting with other elements in the ecosystem. Although it remains a question what comprises an entrepreneurial ecosystem and disentangling its interacting elements is very complex (Alvedalen and Boschma 2017), this study complements the existing studies by developing and testing a conceptual framework that explains the interdependence of the elements with a focus on digital technology. Future studies could refer to the framework to extend the research by examining more interdependencies and causal mechanisms as theorized by Wurth et al. (2021).

Third, the study findings provide new insights into an important but under-researched concept, the national entrepreneurial ecosystem. Since Acs et al. (2014) initiated this concept and provided a framework for analyzing relationships among the system variables, a few studies have documented that cultural norms, formal institutions, government initiatives and policies, and economic dynamics, are important factors that influence the output of the national entrepreneurial ecosystems (Acs, Åstebro, et al. 2016; Autio and Levie 2017; Corrente et al. 2019). However, the influences of the ecosystem elements and their interactions are not confirmed by convincing empirical evidence. This study advances our understanding of the influences and interactions by providing evidence that digital technology and the interactions between digital technology and national culture, formal institutions, and resource endowments produce a high output of the national entrepreneurial ecosystem.

### Policy implications

This study provides several important policy implications. First, our findings imply that increasing the application level of digital technology in a nation helps to promote national entrepreneurship. Policymakers should make instruments to facilitate the development and growth of the digital infrastructure and digitalization in industries. For example, they can undertake steps to encourage the development and application of new technologies, to retrain the current and to train the future workforce for the industry, while also adjusting national educational policies to improve digital competencies (Ilomäki et al. 2016). Second, our findings highlight the importance of interactions between digital technology and national cultural, institutional and economic factors in building a strong entrepreneurial ecosystem. Thus, the measures that promote digitalization alone may not effectively create and reproduce the overall ecosystem. Policymakers should also consider supporting other elements in the system and ensure new policies align with underlying cultural, social, and economic dynamics. For example, while encouraging digitalization, they should accordingly improve institutional quality to reduce the uncertainty facing new ventures, implement instruments to increase the abundance and accessibility of physical resources, and undertake some specific measures to facilitate entrepreneurial activities. Specific to the national entrepreneurial policies and initiatives, digital technology should be considered as one of the factors for creating a fruitful environment for new businesses to stimulate national economies. In summary, our findings help policymakers understand how to look at the role of digital technology from a national entrepreneurial ecosystem perspective.

### Limitation and future research:

First, this study chooses to focus on digital technology and investigate its impact on national entrepreneurship from an entrepreneurial ecosystem perspective. Thus, we only reveal that the interaction between digital technology and other ecosystem elements does enhance the entrepreneurial ecosystem output. Limited by the scope of this study, the more complex interdependences, such as multiple interdependences among the other elements and downward causation (Wurth et al. 2021), are not systematically tested. This leaves the nature of the interdependences and how the interdependences influence the output of the entrepreneurial ecosystem under-researched. Future studies could extend the line of this research by investigating the overall dependence and the output of the system.

Second, this study gives more attention to the quantitative output of the entrepreneurial ecosystem than the qualitative counterpart. Studies have documented that both quantity and quality of entrepreneurship influence economic performance, however, not all entrepreneurial activities contribute equally to economic output (Chowdhury et al. 2019; Sobel 2008). Future studies could investigate the quality-based output of the entrepreneurial ecosystem by incorporating performance-based productive entrepreneurship indicators (Nicotra et al. 2018; Stam 2018), assessing how and by what quality-based and quantity-based output are impacted.

Third, this study incorporates digital technology into the national entrepreneurial ecosystem to analyze the output of the system. However, a more in-depth analysis is needed to understand the relevance of the elements in an entrepreneurial ecosystem. Further theoretical support and expert validation are needed to build a more robust and rigorous structure of a (national) entrepreneurial ecosystem.

Fourth, although this study investigates the entrepreneurial ecosystem with a geographic political/administrative boundary (nation), the spatial and virtual boundaries of an entrepreneurial ecosystem remain under-researched (Audretsch, Cunningham, et al. 2019). Future studies could investigate different types of boundaries to advance the understanding of boundaries, levels and configurations of various entrepreneurial ecosystems. For example, the virtual boundaries determined by people (e.g., Hayter et al. 2018; Wright et al. 2017) or industries are to be investigated.

## Appendix 1

**Table A1:** Robustness test 1 (Digital technology is measured by Percentage of Mobile Users)

	Model 1	Model 2	Model 3	Model 4	Model 5	Model 6	Model 7
Digital		0.002***	0.002***	0.003***	0.002***	0.002***	0.002***
		(0.001)	(0.001)	(0.001)	(0.001)	(0.001)	(0.001)
Capital	0.015***	0.012**	0.012**	0.011**	0.011**	0.012**	0.013***
	(0.005)	(0.005)	(0.005)	(0.005)	(0.005)	(0.005)	(0.005)
Education	-0.078	-0.070	-0.075	-0.068	-0.099	-0.076	-0.037
	(0.072)	(0.072)	(0.072)	(0.070)	(0.072)	(0.071)	(0.071)
Market	-0.017	-0.021	-0.021	-0.022	-0.019	-0.019	-0.015
	(0.027)	(0.027)	(0.027)	(0.026)	(0.026)	(0.026)	(0.026)
Trade	0.005***	0.005***	0.005***	0.003**	0.004***	0.004***	0.004***
	(0.001)	(0.001)	(0.001)	(0.001)	(0.001)	(0.001)	(0.001)
Cultural	0.151**	0.145**	0.142**	0.111*	0.152**	0.144**	0.133*
	(0.068)	(0.068)	(0.069)	(0.067)	(0.068)	(0.068)	(0.068)
Institutional quality	0.001	0.000	-0.000	0.001	-0.001	-0.000	-0.000
	(0.005)	(0.005)	(0.005)	(0.005)	(0.005)	(0.005)	(0.005)
Entrepreneurial policy	-0.025	-0.040	-0.036	-0.036	-0.034	-0.039	-0.047
	(0.034)	(0.034)	(0.035)	(0.034)	(0.034)	(0.034)	(0.034)
Resource accessibility	-0.051	-0.040	-0.034	0.001	-0.026	-0.020	-0.005
	(0.057)	(0.057)	(0.058)	(0.057)	(0.057)	(0.057)	(0.057)
Service industry	0.024***	0.011	0.011	0.006	0.007	0.011	0.015**
	(0.006)	(0.007)	(0.007)	(0.007)	(0.007)	(0.007)	(0.007)
Digital*Cultural			0.001				
			(0.001)				
Digital*Institutional quality				0.000***			
				(0.000)			
Digital*Entrepreneurial policy					0.001***		
					(0.000)		
Digital*Resource accessibility						0.003***	
						(0.001)	
Digital*Service industry							0.000***
							(0.000)
Constant	-2.245***	-1.617**	-1.581**	-1.433**	-1.306**	-1.655***	-2.026***
	(0.583)	(0.629)	(0.631)	(0.614)	(0.635)	(0.625)	(0.630)
Observations	771	763	763	763	763	763	763
R-squared	0.059	0.069	0.070	0.115	0.081	0.083	0.092
Number of code	101	99	99	99	99	99	99
Log likelihood test	-201.5	-197.2	-196.8	-178.1	-192.5	-191.5	-187.8

Standard errors in parentheses

*** p < 0.01, ** p < 0.05, * p < 0.1

## Appendix 2

**Table A2:** Robustness test 2 (Dependent variable is measured by start-up rate)

	Model 1	Model 2	Model 3	Model 4	Model 5	Model 6	Model 7
Digital		0.065***	0.066***	0.078***	0.076***	0.071***	0.061***
		(0.014)	(0.014)	(0.014)	(0.014)	(0.014)	(0.014)
Capital	-0.002	0.011	0.011	0.006	-0.005	0.010	0.018
	(0.045)	(0.044)	(0.044)	(0.044)	(0.044)	(0.044)	(0.044)
Education	-0.811	-0.459	-0.581	-0.450	-0.695	-0.470	-0.371
	(0.661)	(0.662)	(0.665)	(0.658)	(0.666)	(0.659)	(0.662)
Market	-0.180	-0.215	-0.218	-0.205	-0.178	-0.154	-0.222
	(0.245)	(0.239)	(0.239)	(0.238)	(0.239)	(0.239)	(0.239)
Trade	0.018	0.024*	0.025**	0.015	0.022*	0.020*	0.021*
	(0.012)	(0.012)	(0.012)	(0.012)	(0.012)	(0.012)	(0.012)
Cultural	1.355**	0.839	0.702	0.690	0.748	0.613	0.712
	(0.628)	(0.622)	(0.626)	(0.621)	(0.620)	(0.625)	(0.624)
Institutional quality	-0.038	-0.009	-0.017	0.016	-0.007	-0.004	-0.008
	(0.047)	(0.047)	(0.047)	(0.047)	(0.047)	(0.047)	(0.047)
Entrepreneurial policy	-0.365	-0.473	-0.420	-0.489	-0.433	-0.482	-0.467
	(0.311)	(0.314)	(0.315)	(0.312)	(0.313)	(0.312)	(0.313)
Resource accessibility	0.122	0.007	0.045	0.180	0.056	0.060	0.178
	(0.528)	(0.515)	(0.515)	(0.516)	(0.514)	(0.513)	(0.522)
Service industry	0.197***	-0.079	-0.084	-0.146*	-0.139*	-0.097	-0.055
	(0.054)	(0.081)	(0.081)	(0.084)	(0.084)	(0.081)	(0.082)
Digital*Cultural			0.024*				
			(0.014)				
Digital*Institutional quality				0.001***			
				(0.000)			
Digital*Entrepreneurial policy					0.018**		
					(0.007)		
Digital*Resource accessibility						0.042***	
						(0.015)	
Digital*Service industry							0.001*
							(0.001)
Constant	-2.045	10.321*	11.512*	12.165**	14.190**	11.241*	8.169
	(5.388)	(6.090)	(6.121)	(6.091)	(6.257)	(6.070)	(6.176)
Observations	771	755	755	755	755	755	755
R-squared	0.043	0.076	0.080	0.087	0.085	0.086	0.081
Number of code	101	100	100	100	100	100	100
Log likelihood test	-1916	-1850	-1849	-1846	-1847	-1846	-1848

Standard errors in parentheses

*** p < 0.01, ** p < 0.05, * p < 0.1

## Appendix 3

**Table A3:** Robustness test 3 (without United States)

	Model 1	Model 2	Model 3	Model 4	Model 5	Model 6	Model 7
Digital		0.007***	0.007***	0.009***	0.008***	0.007***	0.006***
		(0.001)	(0.001)	(0.002)	(0.002)	(0.001)	(0.001)
Capital	0.014***	0.016***	0.016***	0.015***	0.014***	0.016***	0.017***
	(0.005)	(0.005)	(0.005)	(0.005)	(0.005)	(0.005)	(0.005)
Education	-0.073	-0.052	-0.068	-0.052	-0.084	-0.052	-0.039
	(0.072)	(0.072)	(0.072)	(0.071)	(0.072)	(0.071)	(0.072)
Market	-0.017	-0.019	-0.020	-0.018	-0.014	-0.012	-0.021
	(0.027)	(0.026)	(0.026)	(0.026)	(0.026)	(0.026)	(0.026)
Trade	0.005***	0.005***	0.006***	0.004***	0.005***	0.005***	0.005***
	(0.001)	(0.001)	(0.001)	(0.001)	(0.001)	(0.001)	(0.001)
Cultural	0.142**	0.098	0.081	0.075	0.083	0.069	0.078
	(0.069)	(0.068)	(0.068)	(0.067)	(0.067)	(0.068)	(0.068)
Institutional quality	0.000	0.004	0.003	0.008	0.004	0.005	0.004
	(0.005)	(0.005)	(0.005)	(0.005)	(0.005)	(0.005)	(0.005)
Entrepreneurial policy	-0.015	-0.023	-0.017	-0.026	-0.016	-0.024	-0.023
	(0.034)	(0.034)	(0.034)	(0.034)	(0.034)	(0.034)	(0.034)
Resource accessibility	-0.043	-0.068	-0.064	-0.043	-0.060	-0.059	-0.042
	(0.058)	(0.057)	(0.057)	(0.056)	(0.056)	(0.056)	(0.057)
Service industry	0.023***	-0.004	-0.005	-0.015	-0.013	-0.007	-0.001
	(0.006)	(0.009)	(0.009)	(0.009)	(0.009)	(0.009)	(0.009)
Digital*Cultural			0.003*				
			(0.002)				
Digital*Institutional quality				0.000***			
				(0.000)			
Digital*Entrepreneurial policy					0.003***		
					(0.001)		
Digital*Resource accessibility						0.005***	
						(0.002)	
Digital*Service industry							0.000***
							(0.000)
Constant	-2.159***	-0.989	-0.843	-0.705	-0.437	-0.875	-1.318**
	(0.583)	(0.655)	(0.659)	(0.652)	(0.672)	(0.652)	(0.663)
Observations	755	740	740	740	740	740	740
R-squared	0.057	0.099	0.104	0.122	0.114	0.112	0.110
Number of code	100	99	99	99	99	99	99
Log likelihood test	-197.5	-164.2	-162.2	-154.9	-158	-158.9	-159.7

Standard errors in parentheses

*** p < 0.01, ** p < 0.05, * p < 0.1

## Appendix 4

**Table A4:** Robustness test 4 (add more control variables)

	Model 1	Model 2	Model 3	Model 4	Model 5	Model 6	Model 7
Digital		0.005***	0.005***	0.007***	0.006***	0.006***	0.004***
		(0.002)	(0.002)	(0.002)	(0.002)	(0.002)	(0.002)
Capital	0.014***	0.008	0.009	0.007	0.006	0.009	0.009
	(0.005)	(0.005)	(0.005)	(0.005)	(0.005)	(0.005)	(0.005)
Education	-0.075	-0.039	-0.054	-0.035	-0.071	-0.041	-0.021
	(0.071)	(0.071)	(0.071)	(0.070)	(0.071)	(0.071)	(0.071)
Market	-0.017	-0.014	-0.015	-0.011	-0.008	-0.007	-0.015
	(0.027)	(0.026)	(0.026)	(0.025)	(0.025)	(0.026)	(0.025)
Trade	0.005***	0.005***	0.005***	0.003**	0.005***	0.005***	0.004***
	(0.001)	(0.001)	(0.001)	(0.001)	(0.001)	(0.001)	(0.001)
Cultural	0.153**	0.101	0.086	0.076	0.088	0.077	0.079
	(0.068)	(0.066)	(0.067)	(0.066)	(0.066)	(0.067)	(0.066)
Institutional quality	-0.001	-0.001	-0.002	0.002	-0.001	-0.000	-0.001
	(0.005)	(0.005)	(0.005)	(0.005)	(0.005)	(0.005)	(0.005)
Entrepreneurial policy	-0.021	-0.040	-0.034	-0.044	-0.035	-0.040	-0.040
	(0.034)	(0.034)	(0.034)	(0.033)	(0.034)	(0.034)	(0.034)
Resource accessibility	-0.061	-0.063	-0.059	-0.034	-0.056	-0.059	-0.032
	(0.057)	(0.056)	(0.055)	(0.055)	(0.055)	(0.055)	(0.056)
Service industry	0.023***	0.005	0.004	-0.006	-0.003	0.002	0.010
	(0.006)	(0.009)	(0.009)	(0.009)	(0.009)	(0.009)	(0.009)
d2008	-0.061	-0.051	-0.058	-0.074	-0.059	-0.060	-0.056
	(0.067)	(0.065)	(0.065)	(0.064)	(0.064)	(0.064)	(0.064)
d2009	-0.113**	-0.099*	-0.097*	-0.117**	-0.095*	-0.098*	-0.102*
	(0.058)	(0.056)	(0.056)	(0.055)	(0.056)	(0.056)	(0.056)
Unemployment		-0.022***	-0.021***	-0.025***	-0.023***	-0.021***	-0.024***
		(0.007)	(0.007)	(0.007)	(0.007)	(0.007)	(0.007)
Digital*Cultural			0.003*				
			(0.001)				
Digital*Institutional quality				0.000***			
				(0.000)			
Digital*Entrepreneurial policy					0.003***		
					(0.001)		
Digital*Resource accessibility						0.005***	
						(0.002)	
Digital*Service industry							0.000***
							(0.000)
Constant	-2.061***	-0.772	-0.643	-0.428	-0.235	-0.685	-1.131*
	(0.589)	(0.656)	(0.658)	(0.650)	(0.671)	(0.653)	(0.660)
Observations	771	755	755	755	755	755	755
R-squared	0.065	0.118	0.123	0.146	0.133	0.129	0.132
Number of code	101	100	100	100	100	100	100
Log likelihood test	-198.9	-158.6	-156.5	-146.6	-152.3	-153.8	-152.6

Standard errors in parentheses.

***p < 0.01, **p < 0.05, *p < 0.1.

## Footnotes

1 The focus of the survey “is not only on business characteristics, but also on people’s motivation for starting a business, the actions taken to start and run a business, as well as entrepreneurship related attitudes” https://www.gemconsortium.org/wiki/1599.
